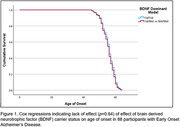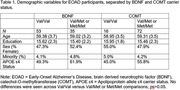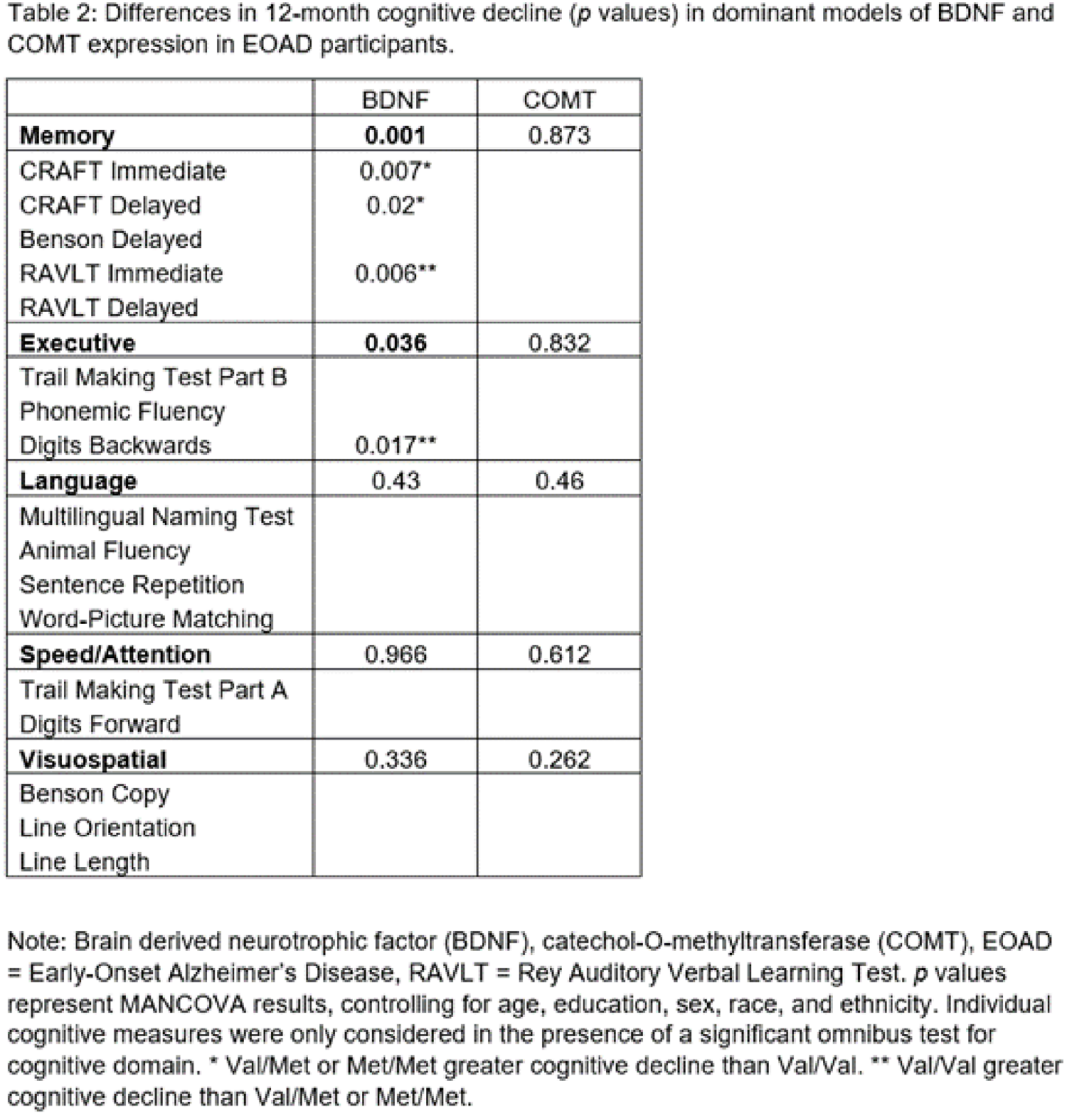# Effects of BDNF and COMT variants on cognitive decline in Early‐Onset Alzheimer’s Disease

**DOI:** 10.1002/alz.092383

**Published:** 2025-01-03

**Authors:** Dustin B. Hammers, Tatiana M. Foroud, Hee Jin Kim, Jane Musema, Jeffrey L. Dage, Ani Eloyan, Maria C. Carrillo, Bradford C. Dickerson, Gil D. Rabinovici, Liana G. Apostolova, Kelly N. Nudelman, LEADS Consortium

**Affiliations:** ^1^ Indiana University School of Medicine, Indianapolis, IN USA; ^2^ Department of Medical and Molecular Genetics, Indiana University School of Medicine, Indianapolis, IN USA; ^3^ Department of Neurology, Indiana University School of Medicine, Indianapolis, IN USA; ^4^ Samsung Medical Center, Sungkyunkwan University School of Medicine, Seoul Korea, Republic of (South); ^5^ Department of Neurology, Indiana School of Medicine, Indianapolis, IN USA; ^6^ Department of Biostatistics, Brown University, Providence, RI USA; ^7^ Alzheimer’s Association, Chicago, IL USA; ^8^ Frontotemporal Disorders Unit and Massachusetts Alzheimer’s Disease Research Center, Department of Neurology, Massachusetts General Hospital and Harvard Medical School, Boston, MA USA; ^9^ Memory and Aging Center, Weill Institute for Neurosciences, University of California, San Francisco, San Francisco, CA USA

## Abstract

**Background:**

Early‐Onset Alzheimer’s Disease (EOAD) is a rare condition that affects only 5% of patients with Alzheimer’s Disease (AD). At present, only basic information is known about the impact of AD risk variants on EOAD, and the effects of more subtle genetic contributions to cognitive decline have yet to be investigated. Genetic variants for brain derived neurotrophic factor (BDNF) and catechol‐O‐methyltransferase (COMT) have both been implicated in cognitive change (Fiocco et al., 2010; Ferrer et al., 2019), consequently the aim of the current study was to examine the role of these genetic variants on cognitive decline in EOAD.

**Method:**

Data from 88 amyloid‐positive EOAD participants enrolled in the Longitudinal Early Onset Alzheimer’s Disease Study (LEADS; aged 40‐64) were analyzed. Exploratory multivariate analyses of covariance (MANCOVA) were conducted to investigate differences in 12‐month cognitive decline as a function of *BDNF* rs6265 (p.V66M) and *COMT* rs4680 (p.V158M) variants using dominant genetic models (Val/Val versus Val/Met or Met/Met). Cox Regression analyses were also conducted to consider the effect of genetic variants on age of onset.

**Result:**

See **Table 1** for demographic characteristics of our sample. MANCOVA, controlling for age, education, sex, and race/ethnicity, showed significant effects for *BDNF* p.V66M on domains of Memory (*p*<0.001) and Executive Functioning (*p* = 0.04; **Table 2**). Specifically, greater 12‐month cognitive decline was observed for the CRAFT Immediate and Delayed Story Memory, with worse performance associated with *BDNF* minor alleles (*p*s. = 0.007 to 0.02). Conversely, worse decline was observed for the reference group for RAVLT Immediate Memory (*p*<0.006) and Digit Span Backwards (*p*<0.02). No significant effects were evident for domains of Language, Speed/Attention, or Visuospatial skills (*p*s = 0.34‐0.97), nor for any analyses of *COMT* carrier status (*p*s = 0.26‐0.87). Cox Regression analyses, controlling for race and ethnicity, were not significant for *BDNF* or *COMT* carrier status (ps = 0.59‐0.64; **Figure 1**).

**Conclusion:**

Results suggest subtle effects of *BDNF* p.V66M carrier status on memory decline in EOAD participants, which was not observed for disease progression/age‐of‐onset. No effects for *COMT* p.V158M carrier status were observed. Future investigation will replicate these effects in larger samples, permitting stratification of additional covariates including *APOE* genotype.